# Knockdown of GGCT inhibits cell proliferation and induces late apoptosis in human gastric cancer

**DOI:** 10.1186/s12858-016-0075-8

**Published:** 2016-12-01

**Authors:** Wenjie Zhang, Lei Chen, Honggang Xiang, Chunhua Hu, Weibin Shi, Ping Dong, Wenjie Lv

**Affiliations:** Department of General Surgery, Xinhua Hospital Affiliated to Shanghai Jiao Tong University, Shanghai, China

**Keywords:** Gastric cancer, GGCT, shRNA, Proliferation, Apoptosis

## Abstract

**Background:**

Gamma glutamylcyclotransferase (GGCT) has been proved to be involved in various cancers, but the biological function of GGCT in gastric cancer is still largely unknown.

**Methods:**

The expression level of GGCT was evaluated by informatics analyses based on the Oncomine database. GGCT gene was then effectively knocked down via lentivirus mediated short hairpin RNA (shRNA) system. Then a series of functional assays, including MTT, colony formation and flow cytometry analysis were conducted on gastric cancer cells following GGCT knockdown.

**Results:**

We found GGCT is commonly up-regulated in gastric cancer tissues. Furthermore, MTT analysis showed that GGCT depletion significantly inhibited cell proliferation in MGC80-3 and AGS cells. Colony formation assay revealed that depletion of GGCT reduced the colony formation ability in gastric cancer cells. What’s more, cell cycle analysis showed that depletion of GGCT induced gastric cancer cell cycle arrested G2/M phase. More importantly, cell apoptosis analysis further revealed that GGCT inhibition induced early and late cell apoptosis in gastric cancer.

**Conclusion:**

This study suggests GGCT is essential for gastric cancer proliferation and its downregulation may provide a potential anticancer therapy for gastric cancer.

## Background

Gastric cancer, as a common cancer, has the third highest mortality rate in both sexes worldwide, especially in developing countries and regions [1]. However, lack of effective early diagnostic methods makes gastric cancer usually be diagnosed at late stages and missed the best treatment time for curative surgery and radiotherapy [2, 3]. Meanwhile, chemotherapy usually causes severe side effects, such as nausea, vomit, white blood cell reduction etc., and thus is not suitable for elder or weak patients. Hence, early diagnosis and targeted therapy are critical for better prognosis of gastric cancer in the future. It has been reported that multiple gene abnormalities led to the initiation and development of gastric cancer [4]. Thus, investigating the underling mechanisms and identifying key genes of gastric cancer will provide novel evidences for early diagnosis and targeted therapy against gastric cancer.

Gamma glutamyl cyclotransferase/glutamyl cyclotransferase-γ (GGCT), as a critical enzyme in the regulation of a γ-glutamyl cycle via mediation of glutathione degradation, can specifically convert γ-Glu-AA into pyroglutamate (pyroGlu) [5]. It has been reported that GGCT was strongly accumulated in various cancers, including breast, bladder, esophagus, stomach and lung cancers [6–12]. Knockdown of GGCT can suppress cell proliferation ability in bladder cancer cells [13] and induced the cell cytotoxicity against MCF-7/ADR cells in vitro and in vivo [13]. Taken together, GGCT was overexpressed in many cancer cells, including gastric cancer. However, the underling mechanism of how GGCT regulates gastric cancer remains elusive.

To explore the biological function and its underlying mechanism of GGCT in human gastric cancer, the present study firstly confirmed that the expression level of GGCT is highly in gastric cancer tissues by analyzing data derived from Oncomine database. Knockdown of GGCT with the shRNA system significantly suppressed cell features, including cell proliferation, colony formation ability in gastric cancer cells. Furthermore, we found that depletion of GGCT promotes late apoptosis and induced gastric cancer cells to be arrested in sub-G1 and G2/M phases. These results indicated that GGCT plays an important role in cell proliferation and progression of gastric cancer.

## Methods

### Cell lines and cell culture

Human gastric cancer cells (MGC80-3and AGS), and human embryonic kidney 293T (HEK293T) cell lines were purchased from the Cell Bank of Chinese Academy of Science (Shanghai, China). MGC80-3 and AGS cells were maintained in RPMI 1640 medium (Hyclone, Cat no. SH30809.01B) with 10% fetal bovine serum (FBS, Cat no. 04-001-1A-1351574). HEK293T cells were cultured in DMEM medium (Hyclone, Cat no. SH30243.01B+) with 10% FBS. All of the cells were maintained at 37 °C in a humidified atmosphere with 5% CO_2._

### Oncomine database analysis

The informatics data on GGCT mRNA expression in stomach cancers were obtained from the Oncominedatabase (https://www.oncomine.org). Wang et al. (GEO accession GSE19826) [14] and Cui et al. (GEO accession GSE27342) [15] datasets were used to compared expression levels of GGCT between gastric cancer and normal gastric tissues according to the standard procedures as previously described [16].

### Interference vector construction and infection

The shRNA oligos for GGCT gene knockdown were designed and cloned into the lentiviral expression vector (pFH-L, Shanghai Hollybio, China) between restriction sites *NheI* and *PacI*. The sequence of shRNA targeting GGCT was 5′-GATTATTTGCATGGGTGCAAACTCGAGTTTGCACCCATGCAAATAATCTTTTTT-3′and the scramblesequence5′-TTCTCCGAACGTGTCACGT-3′ was used as negative control. The two recombinant vectors were designated Lenti-shGGCT and Lenti-shCon, respectively. These plasmid DNAs were transfected into the *E.coli* DH5α competent cells and then the cells were purified with the endotox in-free plasmid purification kit (Qaigen, CA, USA). PCR and sequencing analyses were conducted to confirm the successful ligation. These shRNA vectors and packaging pHelper plasmids (pVSVG-I and pCMV△R8.92) (Shanghai Hollybio, China) were co-transfected into HEK293T cells. At 96 h after transfection, supernatant was collected and centrifuged to obtain the lentiviruses. The titer of lentiviruses was determined through counting Green Fluorescent Protein (GFP)-positive cells under a fluorescence microscope (Olympus, Tokyo, Japan). Titer was calculated as IU/ml = (the numbers of GFP-positive cells) × (dilution factor)/(volume of virus solution). MGC80-3 and AGS cells were infected with the viruses at the MOI of 60 and mock-infected cells were used as negative control.

### Quantitative RT-PCR (qRT-PCR) analysis

MGC80-3 and AGS cells were harvested at 5 days post infection. Total RNA of the cells was extracted with TRIzol® reagent (Invitrogen, Cat no.15596-018) separately. The purity and integrity of total RNA was assessed by spectrophotometry and agarose gel electrophoreshs, respectively. First-strand cDNA was then synthesized with 1 μg RNA using the Reverse Transcription System (BioRad, CA, USA). The sequences of qRT-PCR primers were as follows: 5′-CAGCAACCTGCTGACAGAGA-3′ (forward) and 5′-CCCTTCTTGCTCATCCAGAG-3′ (reverse) for GGCT,5′-GTGGACATCCGCAAAGAC-3′ (forward) and 5′-AAAGGGTGTAACGCAACTA-3′ (reverse) for Actin. PCR reaction was carried out through with BioRad Connet Real-Time PCR platform. And the conditions were 1 min at 95 °C, 40 cycles of 95 °C for 5 s, and 60 °C for 20 s, read absorbance value at the extension stage.

### Western blot

MGC80-3 and AGS cells were harvested at the 5^th^ day post infection. Then cells were lysed in 2× SDS Sample Buffer (100 mM Tris-HCl, pH = 6.8, 10 mM EDTA, 4% SDS, 10% Glycine). The concentration of protein in the cell lysate was determined using the BCA protein assay kit (Pierce Biotechnology, Cat no. 23235). Totally, 30 μg of protein was loaded on per lane, followed by separation using SDS-PAGE, and transfer to PVDF membrane. Then the membrane was subsequently incubated with primary antibodies, including rabbit anti-human-GGCT (Sigma, Cat no. HPA020735, 1:500), rabbit anti-human-GAPDH (Proteintech Group, Inc., Cat no. 10494-1-AP, 1:60000), overnight at 4 °C, which was followed by incubation with secondary antibody, horseradish peroxidase-conjugated anti-rabbit IgG (Santa Cruz, Cat no.SC-2054, 1:5000), for 1 h at room temperature. Blots were visualized using ECL Test Kit (Amersiam, Cat no. RPN2132). GAPDH was used as the internal control. Density analysis was carried out using Quantity One software (BioRad).

### Cell proliferation and colony formation assays

The cell viability of MGC80-3 and AGS cells before and after infection was tested using the MTT assay. MGC80-3 and AGS cells were plated at a density of 2000 cells/well in a 96-well plate after 4 days of lentivirus infection. For MTT assay, MTT solution was added into each well every day of the next 5 days and incubated at 37 °C in humidified atmosphere with 5% CO_2_ for 4 h, and then 150 μl acidic isopropanol (0.01 M HCl, 10% SDS, 5% isopropanol) was added. Absorbance values at 595 nm were measured with Epoch Microplate Spectrophotometer (Biotek, CA, USA).

To judge colony formation abilities of the cells, transfected MGC80-3 cells were seeded in a 6-well plate at the concentration of 400 cells/well after 4 days of infection. Cells were fixed with methanol and, stained with 1% crystal violet (Beyotime Cat no. C0121) for 10 min, then gently washed with water and dried in air. Visible colonies containing more than 50 cells were manually counted under fluorescence microscopy. Image analysis was performed using Image-Pro®PlusVersion 6.0 (Media Cybernetics, MD, USA).

### Cell cycle analysis

Cell cycle distribution was analyzed using PI (propidium iodide) following the manufacturer’s instructions. Briefly, MGC80-3 cells were seeded in 6 cm dishes at 5 × 10^4^ cells/dish 4 days after lentivirus infection. After 6 days of cultivation, cells were washed and suspended in PBS containing 50 μg/ml RNase A (Sigma-Aldrich) and 50 μg/ml PI for 1 h in the dark. Fluorescence associated cellsorting (FACS) assay with FACSCalibur (Beckman Coulter, CA, USA) was used to count cell populations in each cell cycle phases and the results were analyzed with the ModFit (Verity Software House, Maine, USA) software.

### Apoptosis assay

MGC80-3 cells (1 × 10^5^/well) were collected after 48 h of transfection with Lenti-shGGCT or Lenti-shCon and stained with both Annexin V (allophycocyanin [APC] conjugated) and Propidium Iodide (PI) according to the manufacturer’s instructions (BD Biosciences, Erembodegem, Belgium). Apoptosis was detected by flow cytometry (BD FACSCalibur).

### Statistical analysis

The results were expressed as the mean ± SD. Statistics were analyzed using SPSS software version 13.0. The student’s *t*-test was used to evaluate the difference between Lenti-shGGCT and Lenti-shCon group. The *p*-value < 0.05 was considered as statistically significant.

## Results

### GGCT mRNA was overexpressed in gastric cancer

**Fig. 1:**
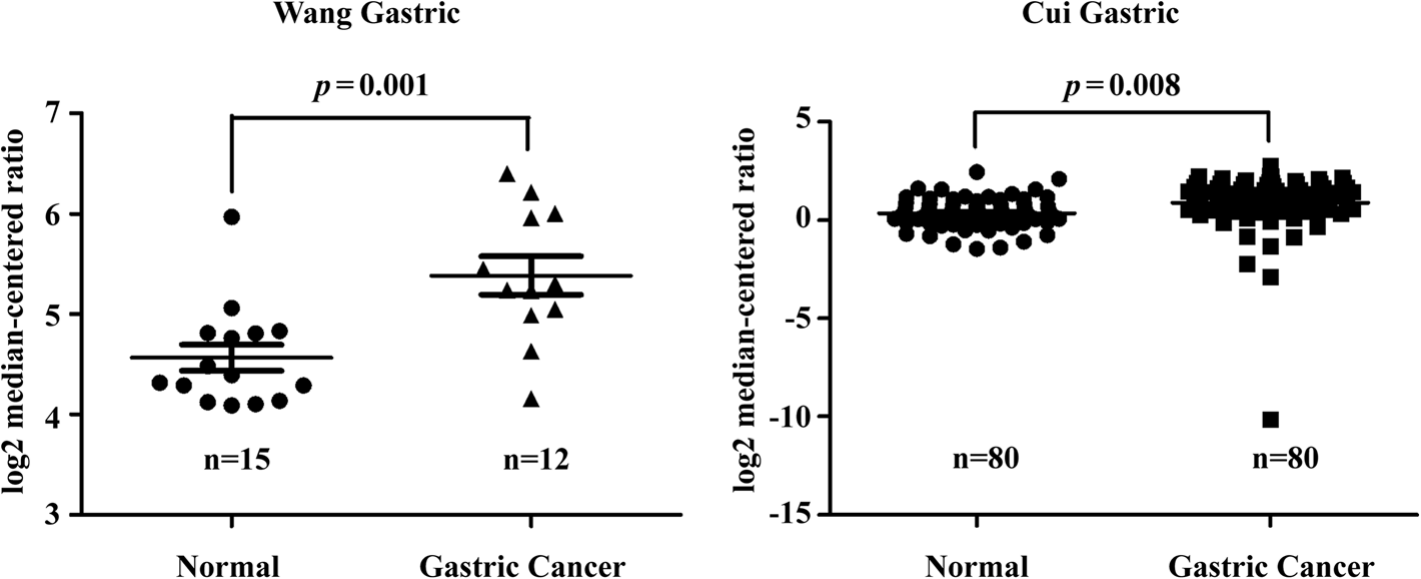
GGCT is up-regulated in Oncomine database analysis. Integrative analysis of GGCT mRNA expression levels in gastric cancer vs. normal gastric type. A box plot shows relative mRNA expression level of GGCT in Wang and Cui datasets grouped by gastric cancer and normal tissue, respectively. *p* value indicates the significant difference between two groups

As GGCT has been reported to be upregulated in several cancers, we conducted a review of previous microarray data for the expression of GGCT on mRNA level in human gastric cancers. Results of searches in Oncomine database (Fig. 1) revealed that expression of GGCT was significantly increased in human gastric cancer as compared with healthy gastric tissues as shown in Wang (*p* = 0.001) and Cui datasets (*p* = 0.008).

### Lenti-shGGCT reduces GGCT expression in human gastric cancer MGC80-3 and AGS cells

**Fig. 2:**
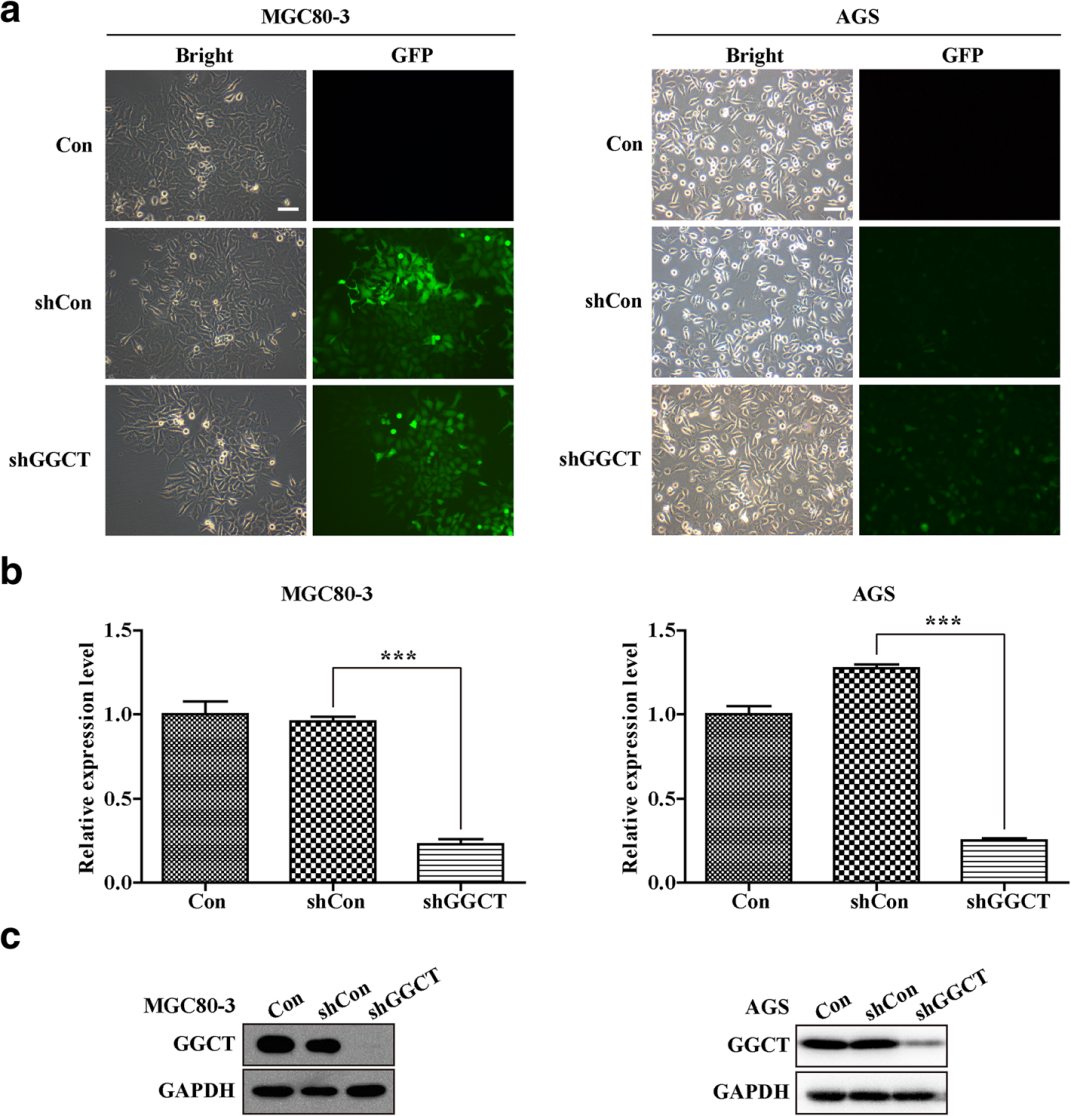
Lentivirus-mediated shRNA specifically reduced GGCT expression in MGC80-3 and AGS cells. **a** Green fluorescent images of MGC80-3 cells under a 100× magnification microscope and under inverted fluorescent microscope at 96 h after lentivirus infection, showing the successful infection of lentivirus expressing Lenti-shGGCT, Lenti-shCon, or Con, Scale bars, 10 μm. **b** The mRNA levels of GGCT in MGC80-3 and AGS cells after lentivirus infection analyzed by qRT-PCR. **c** GGCT protein levels in MGC80-3 and AGS cells after lentivirus infection analyzed by Western blot. ***p < 0.001

To explore the role GGCT plays in human gastric cancer, a lentiviral vector system expressing shRNA against GGCT was constructed in MGC80-3 and AGS cells. GFP was applied as a reporter gene. The high rate of GFP-positive cells was observed by fluorescence microscopy at 96 h post-infection (Fig. 2a), which suggests the high efficiency of infection. The GGCT knock-down efficiency was further validated at both mRNA and protein levels. As Fig. 2b and c display, the relative mRNA and protein expression of GGCT (*p* < 0.001) were significantly decreased in the MGC80-3and AGS cells infected by Lenti-shGGCT, compared with the cells infected with Lenti-shCon and the control cells. Those results indicated that the Lenti-shRNA infection system exerted powerful and specific knock-down effects on GGCT expression in MGC80-3 and AGS cells.

### Lenti-shGGCT suppressed the viability and proliferation of MGC80-3 and AGS cells

**Fig. 3:**
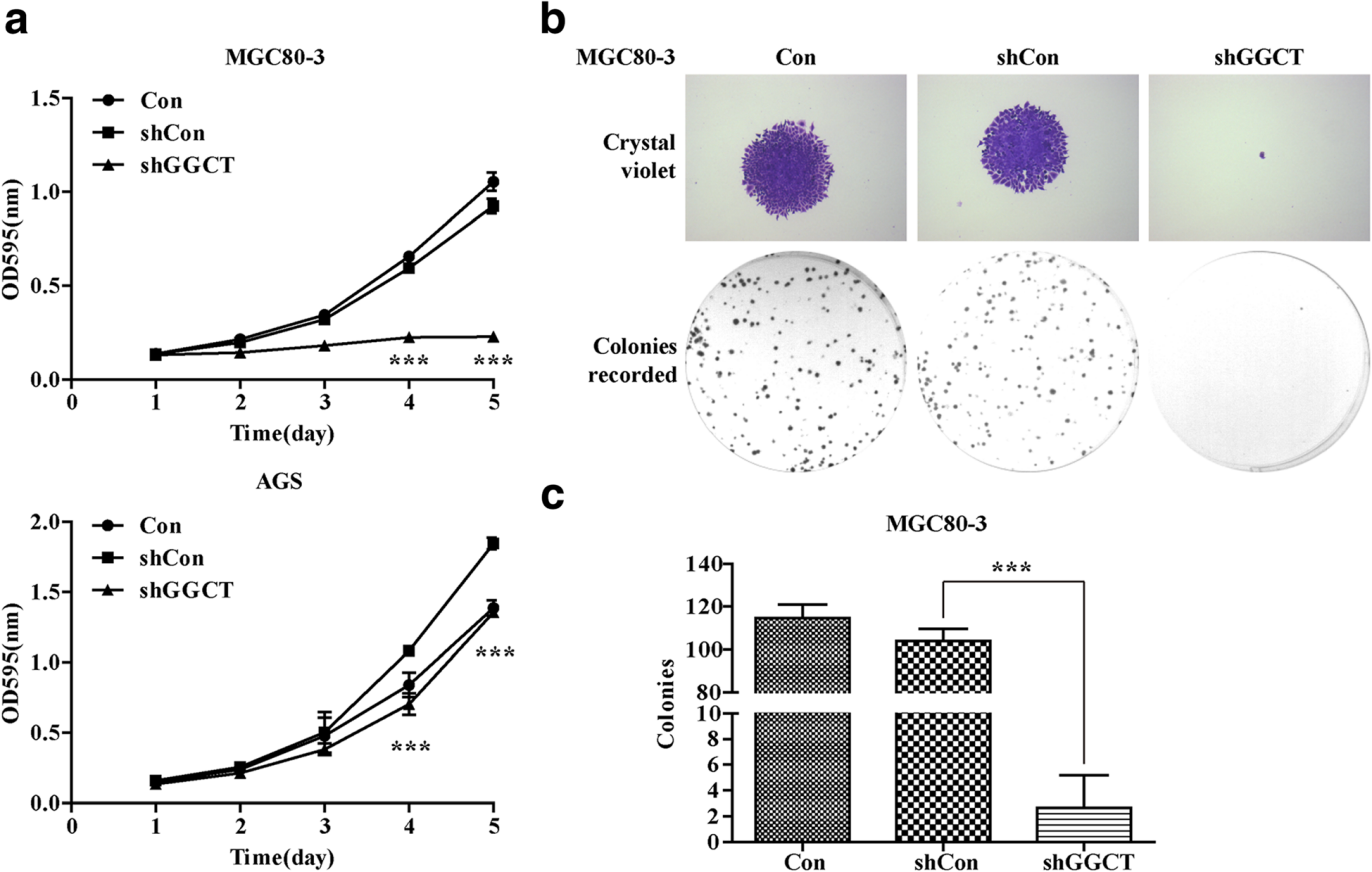
Knockdown of GGCT inhibited the viability and proliferation of MGC80-3 and AGS cells. **a** The growth curve of MGC80-3 and AGS cells with different treatments (Con, Lenti-shCon, or Lenti-shGGCT) assessed by MTT assay. **b** Images of MGC80-3 cells recorded under micro and macro view, representing the size and the number of colonies in each group of cells. **c** Statistical analysis for the number of colonies formed in MGC80-3 cells with different treatments (Con, Lenti-shCon, or Lenti-shGGCT). All data represented as mean ± SD from 3 independent experiments. ***p < 0.001

To discover the potential functions of GGCT in gastric cancer, the cell proliferation assay was conducted. The Lenti-shCon and Lenti-shGGCT infected MGC80-3 and AGS cells were seeded on 96-well plates for a MTT assay in the following 5 days. Interestingly, the depletion of GGCT significantly attenuated the proliferation ability of MGC80-3 and AGS cells, as compared with that in Lenti-shCon infected and control cells (Fig. 3a, *p* < 0.001). In addition, colony formation plays a critical role in the development of tumor in vivo. To study whether GGCT depletion affected the clonogenic potential of gastric cancer cells, the colony formation assay was performed in MGC80-3 cells. As Lenti-shGGCT infected MGC80-3 cells exhibited a complete GGCT depletion, it resulted in better performance in proliferation assay as compared with that in AGS cells. Notably, much less colonies formed in Lenti-shGGCT infected MGC80-3 cells, compared toMGC80-3 cells infected with Lenti-shCon (*p* < 0.001) (Fig. 3b and c). These results indicated that GGCT depletion significantly slowed cell proliferation and suppressed colony formation potential of MGC80-3 cells.

### GGCT depletion caused cell cycle arrest in MGC80-3 cells

**Fig. 4:**
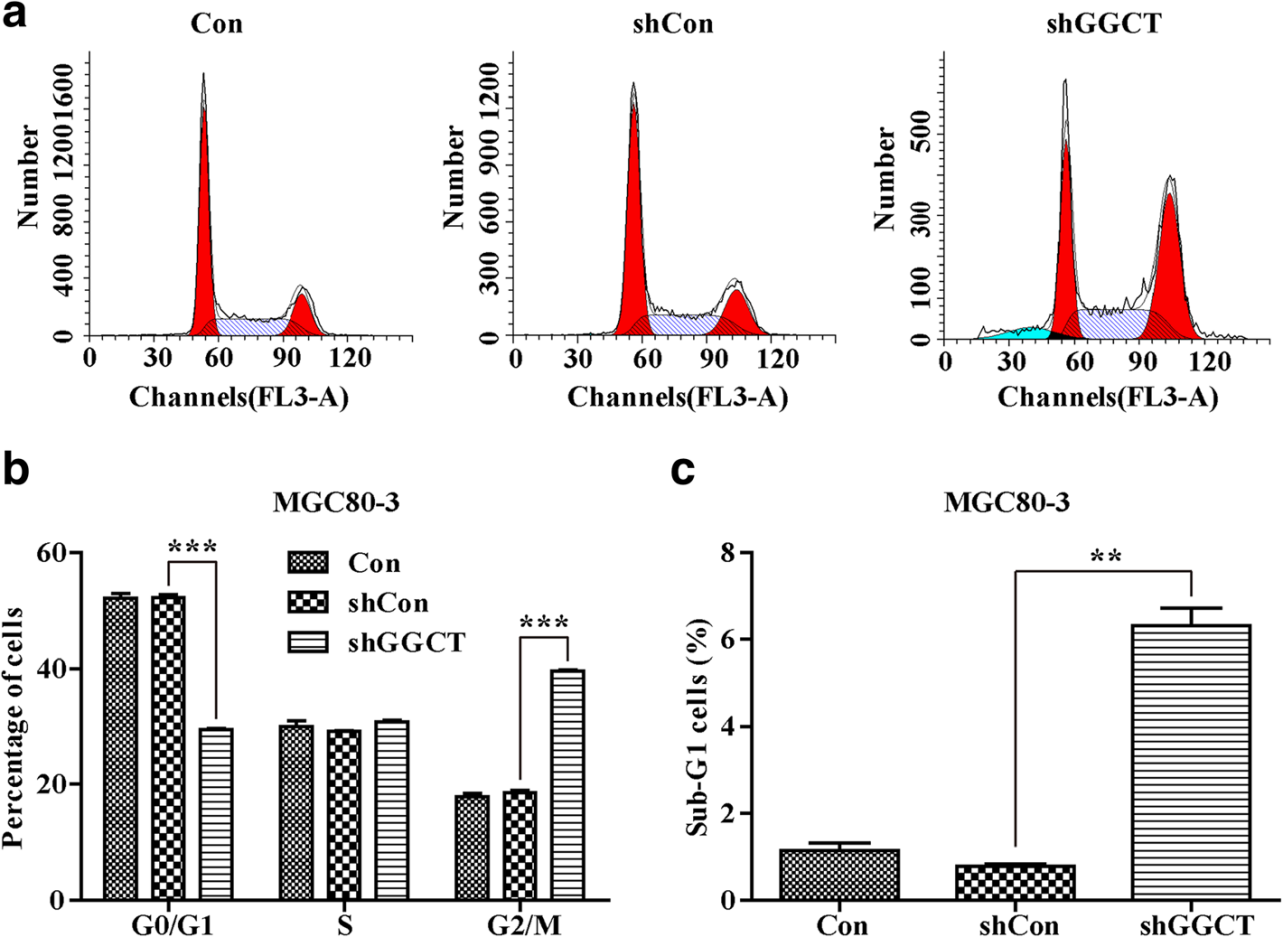
Knockdown of GGCT induced cell cycle arrest during G2/M and Sub-G1phase in MGC80-3 cells. **a** FACS analysis of cell cycle distribution in MGC80-3 cells. A hypodiploid apex representing the apoptotic cells was found before the G1apex in Lenti-shGGCT infected MGC80-3 cells. **b** Down-regulation of GGCT in MGC80-3cells led to an increase of cells at G2/M phase and concomitantly a decrease of cells at G0/G1 phase. **c** Down-regulation of GGCT in MGC80-3 cells led to an increase of cells at Sub-G1 phase. Data represents mean ± SD from 3 independent experiments. ** *p* < 0.01, ***p < 0.001

To identify the mechanism underling the anti-proliferation effect of GGCT depletion, cell cycle distribution of shGGCT-infected MGC80-3 cells was analyzed by flow cytometry (Fig. 4a). There was a significant reduction of cell number of Lenti-shGGCT infected MGC80-3 cells in G0/G1 phase and a corresponding increase in G2/M phase, compared with Lenti-shCon infected and control cells (Fig. 4b). In addition, infection of lenti-shGGCT resulted in a remarkable increase of MGC80-3 cell number in the sub-G1 phase (Fig. 4c) suggesting that GGCT depletion might induce cell apoptosis.

### GGCT depletion caused cell apoptosis

**Fig. 5:**
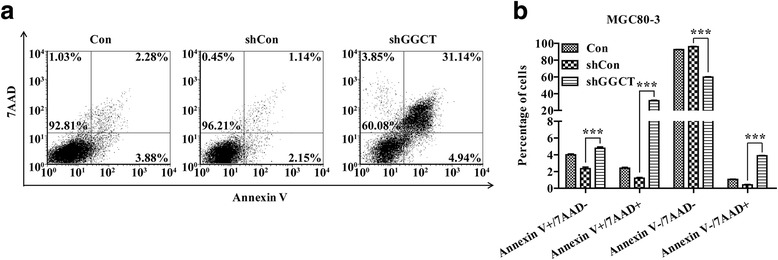
Knockdownof GGCT induced cell apoptosis in MGC80-3 cells. Apoptosis cells were assessed by Annexin V/PI double staining and then were subjected to flow cytometry analysis. **a** Representative images showing Annexin V/PI staining results in MGC80-3 cells with different treatments (Con, Lenti-shCon, or Lv-sh GGCT). **b** Statistical analysis of the apoptosis assay. **p < 0.01; ***p < 0.001. The experiment was performed in triplicates

In order to determine the effects of GGCT depletion on apoptosis of gastric cancer cell, Annexin V/PI double staining was performed on MGC80-3 cells. Interestingly, after observing Annexin V/PI double positive cells, it was found that depletion of GGCT largely promoted late apoptosis (Fig. 5a). Statistical analysis revealed that approximately 30-fold and 15-foldincrease of late apoptotic populations was detected in Lenti-shGGCT-infected MGC80-3 cells (31.4% ± 0.49%), as compared to Lenti-shCon-infected and control MGC80-3 cells (1.14% ± 0.08%, 2.28% ± 0.10%), respectively (Fig. 5b, *p* < 0.001). These results indicated downregulation of GGCT could promote cell apoptosis in gastric cancer cells.

## Discussion

Gastric cancer is one of the most common causes of cancer-related death worldwide as usually be diagnosed at late stages and missed the best treatment time [2, 3]. Thus, early diagnosis and targeted therapy are critical for better prognosis of gastric cancer. To investigate the underling mechanisms and identify key molecular markers will provide novel evidences for early diagnosis and targeted therapy against gastric cancer. GGCT is a critical enzyme that regulates the biological processes of glutathione, which is the major antioxidant in cells. Herein, we identified GGCT was expressedhigher in human gastric cancer tissues than normal tissues. Knockdown of GGCT weakened cell proliferation and colony formation abilities of gastric cancer cells via increasing cell cycle arrest and inducting late apoptosis. These findings not only clarify the effects of GGCT depletion on gaistric cancer, but also provide new evidence and basis for new methodsof early diagnosisand target treatment of gastric cancer.

Recently, GGCT has been found strongly accumulated in various cancers, including breast, bladder, esophagus, stomach and lung cancers [6–12]. Based on theanalysis of two independent studies in Oncomine database, we found that GGCT is highly accumulated in gastric cancer cells. Furthermore, it has beenproved thatGGCTfunctioned as a novel biomarker of esophageal squamous malignancies, as its expression levels strongly correlated with lymph node metastasis and differentiation degree [6–11], in consistent with that overexpression and depletion of GGCT resulted in proliferative and antiproliferative activities in bladder cancer cells, respectively [9]. Here, we observed that GGCT depletion could remarkably inhibit cell proliferation and colony formation activities ofhuman gastric cancer cells.

Furthermore, results of cell cycle and apoptosis analysis revealed that GGCT inhibition negatively regulated gastric cancer progression via induction of cell arrest and late apoptosis in MGC80-3 cells, in consistent withthe study of GGCT in lung cancer [17]. In previous studies, it wasindicatedthat knockdown of GGCT weakened proliferation and colony formation abilities of lung cancer cells through G0/G1 phase arrest. Moreover, the expression levels of G0/G1-associated markers, CDK4, CDK6, and cyclin D1, declinedand the expression level of cleaved PARP was obviously increased in GGCT knockdown cells [17]. Ohno et al. found that GGCT is closely associated with cell cycle [9], which was consistent with our finding that depeletion of GGCT arrested MGC80-3 gastric cancer cells in the sub-G1 and G2/M phases, suggesting that knockdown of GGCT inhibited cell phase transition and suppressed cell proliferation. Whether GGCT suppress cell proliferation through common cell cycle realted molecules, like cyclins and cdks, will provide deeper understanding of the mechanism and needs further investigation.

Furthermore, to alter gene expression in cells, there were various methods, such as RNAi with shRNA or siRNA. This study has generated efficient and stable GGCT depletion cell lines using shRNA-based GGCT knock-down technique instead of siRNA. AlthoughsiRNA has been widely used as a therapeutic tool [18], administration of siRNA often have problems of poor stability, low transfection efficiency, and lowtissue penetration. The shRNA-based GGCT depletion provides a more stable and reliabe method for lab research use [19].

## Conclusion

The present study statedthat GGCT was upregulated in human gastric cancer tissues. Knockdown of GGCT inhibits cell proliferation and colony formation via increasing cell cycle arrest and inducting late apoptosis. These findings not only clarify the effects of GGCT depletion on gaistric cancer, but also provide new evidence for development of early diagnosis and targeted therapies of human gastric cancer. However, what is the potential correlation between the dysregulated cycle of glutathione and cell division, and whether downregulation of GGCT interferes with the transportation of amino acids leading to cell division disruption in gastric cancer need further investigation.
